# Influence of Risk Factors on the Well-Being of Elderly Women with Knee Osteoarthritis

**DOI:** 10.3390/medicina59081396

**Published:** 2023-07-29

**Authors:** Ivana Minaković, Jelena Zvekić Svorcan, Tanja Janković, Hajdana Glomazić, Mirjana Smuđa, Dejan Živanović, Jovan Javorac, Bela Kolarš

**Affiliations:** 1 Faculty of Medicine, University of Novi Sad, 21000 Novi Sad, Serbia; jelena.zvekic-svorcan@mf.uns.ac.rs (J.Z.S.); tanja.jankovic@mf.uns.ac.rs (T.J.); mirjana.smudja@uns.ac.rs (M.S.); dejan.zivanovic@mf.uns.ac.rs (D.Ž.); jovan.javorac@mf.uns.ac.rs (J.J.); 902009d22@mf.uns.ac.rs (B.K.); 2 Health Center “Novi Sad”, 21000 Novi Sad, Serbia; 3 Special Hospital for Rheumatic Diseases Novi Sad, 21000 Novi Sad, Serbia; 4 Institute of Criminological and Sociological Research, 11000 Belgrade, Serbia; hajdana.ng@gmail.com; 5 Department of Higher Medical School, The Academy of Applied Studies Belgrade, 11000 Belgrade, Serbia; 6 Department of Psychology, College of Social Work, 11000 Belgrade, Serbia; 7 Institute for Pulmonary Diseases of Vojvodina, 21000 Novi Sad, Serbia

**Keywords:** osteoarthritis, knee, predictors, social functioning, pain, physical function, mental health

## Abstract

*Background and Objectives:* Knee osteoarthritis (KOA) is a widespread chronic joint disease characterized by functional limitations and pain. Functioning restrictions exert a detrimental impact on societal integration, relationships, and psychological well-being, resulting in significant emotional distress in KOA patients. The objective of this study is to examine how various risk factors impact the emotional well-being of individuals with KOA. *Materials and Methods:* This prospective cross-sectional study involved 154 postmenopausal women treated at the Special Hospital for Rheumatic Diseases in Novi Sad, Serbia. The experimental group comprised 97 individuals with chronic knee pain and structural knee damage (Kellgren–Lawrence (KL) scale II-IV), while the control group had 53 individuals with chronic knee pain but no structural knee damage (KL scale 0-I). The collected data consisted of sociodemographic factors, general characteristics, associated diseases, and laboratory results. Adequate anthropometric measurements were conducted, and all subjects were required to complete the SF-36 RAND questionnaire. *Results:* The analysis identified several variables that independently influenced emotional well-being. These included pain intensity (beta (β) 0.21; 95% CI: 0.03–0.20; *p* < 0.01), social functioning (beta (β) 0.47; 95% CI: 0.23–0.43; *p* < 0.001), physical functioning (beta (β) 0.23; 95% CI: 0.04–0.21; *p* < 0.01), and education level (8–12 years: beta (β) 0.25; 95% CI: 1.47–9.41; *p* < 0.01; >12 years: beta (β) 0.27; 95% CI: 2.51–12.67; *p* < 0.01). However, the multivariate model revealed that only social functioning (beta (β) 0.57; 95% CI: 0.27–0.53; *p* < 0.001) and education level (8–12 years: beta (β) 0.21; 95% CI: 1.10–8.260; *p* < 0.05; >12 years: beta (β) 0.21; 95% CI: 1.18–10.30; *p* < 0.05) were significantly associated with emotional well-being in KOA patients. *Conclusions:* The findings of this study indicate that a reduced social functioning and a lower educational attainment are linked to a poorer emotional well-being among patients with KOA.

## 1. Introduction

Osteoarthritis (OA) is a diverse condition with various phenotypes [1]. It can affect any joint in the human body, although it primarily affects peripheral joints such as the knee, hip, hand, and spine [2,3]. The global prevalence of OA is approximately 350 million people [4], and it is commonly observed in one out of every three individuals over the age of 65 [5,6]. This disease is more prevalent among women, and obese women face a significant lifetime risk of up to 23.8% for symptomatic KOA [1]. Given its widespread occurrence, OA not only affects the individual’s health, but also has a substantial impact on the overall population’s health [1,7].

The presence of structural and functional limitations in patients with OA significantly contributes to pain and disability [8,9,10]. These symptoms are widely recognized as key manifestations of the disease [9,11]. Moreover, individuals with OA experience a considerable decline in their quality of life (QOL) due to diminished social connections, relationships, and emotional well-being [8]. Consequently, there is an increased risk of developing mental disorders [10,12]. These findings are consistent with a multitude of epidemiological studies, indicating a robust interconnection between physical diseases and common mental health conditions. They exhibit a high co-morbidity, meaning they often co-occur in individuals, and share crucial pathways that contribute to the development of ill health and disease [13].

In general, health is commonly perceived as the absence of illness or disease. These notions have been embedded in healthcare models, which tend to focus only on alleviating impairment and distress [13]. Presently, there are various treatment choices for KOA, including behavioral modifications like weight loss, exercise, and physical therapy coupled with pain-relieving medications, as well as surgical interventions [9]. Although numerous options are accessible, certain patients may still encounter insufficient symptom management, while others might experience adverse effects from the available interventions [14]. Among the surgical options, orthopedic surgery with total arthroplasty is recognized as a highly effective approach for addressing KOA [15]. However, it is essential to note that the surgery still has a failure rate that can affect the patient’s psychology [16].

The impact of OA on mental health is particularly evident in the heightened vulnerability to conditions such as depression [1,12] and anxiety disorders [17]. This aspect is of the utmost significance since poor mental health, particularly the presence of depression, has been linked to unfavorable treatment outcomes in OA patients [12,18], as well as increasing the perception of pain [9]. Given that well-being encompasses various dimensions, including mental, physical, social, and environmental aspects [17,19], it becomes evident that placing a greater emphasis on providing psychological support to OA patients is crucial not only for pain reduction and functional improvement, but also for overall health enhancement.

Nevertheless, there is a scarcity of research regarding the factors that predict emotional well-being in patients with KOA. The aim of this study was to identify these predictors, thereby allowing for personalized patient treatment tailored to their specific characteristics, with a focus on improving modifiable risk factors.

## 2. Materials and Methods

### 2.1. Participants

A prospective cross-sectional study involved 154 postmenopausal women who were receiving treatment for chronic knee pain at the Special Hospital for Rheumatic Diseases Novi Sad, Serbia, between February 2022 and March 2023. The study was conducted in accordance with the guidelines of the Declaration of Helsinki. The study protocol obtained approval from the Ethics Committee of the Special Hospital for Rheumatic Diseases Novi Sad, Serbia (approval number: 14/29–3/1–21) and the Ethics Committee of the Faculty of Medicine Novi Sad, Serbia (approval number: 01–39/109/1). The informed consent was obtained from all participants.

Inclusion criteria: Women in postmenopause, aged 60–75 years who reported knee pain of intensity ≥ 3 on the numeric rating scale (NRS) persisting for a minimum duration of 3 months. Exclusion criteria: Participants with a history of knee surgery or injury, as well as presence of inflammatory rheumatic disease, metabolic joint diseases (such as gout and chondrocalcinosis), neuromuscular diseases (such as muscular dystrophies and myasthenia gravis) and participants who are currently being treated for a malignant disease. Furthermore, patients undergoing physical therapy, receiving corticosteroid therapy, or undergoing intraarticular chondroprotective therapy within the past three and six months, respectively, were also excluded.

At the outset, the study comprised 154 subjects, but four of them were later excluded upon the diagnosis of inflammatory rheumatic disease.

All patients suffering from chronic knee pain, who were referred to the Special Hospital for Rheumatic Diseases in Novi Sad, underwent an initial examination conducted by a physical medicine specialist. In the first phase of the study, sociodemographic information and data concerning related medical conditions were gathered, and all participants completed the SF-36-RAND questionnaire [20]. Body mass and height were measured using a precise digital weight scale and an adult height scale, respectively. Obesity level was determined by calculating the body mass index (BMI), which was obtained by dividing weight in kilograms by height in square meters. Waist circumference was measured with a measuring tape, and knee range of motion (ROM) was assessed using a goniometer. A knee flexion ≥ 110 and an extension of 0 were considered as satisfactory ROM [21]. Blood pressure was assessed while the individual was in a seated and relaxed position, using a validated and calibrated Riester sphygmomanometer on both hands. After a short interval, the measurement was repeated on the hand that initially showed the higher pressure reading [22]. In the second phase of the study, all participants underwent bilateral anteroposterior and lateral X-rays of both knees. A skilled radiologist assessed the degree of radiological damage using both the Kellgren–Lawrence (KL) scale and the Altman atlas. Subsequently, the subjects were classified into two groups based on these evaluations. The experimental group consisted of 97 subjects who showed radiological damage classified as grade II-IV on the KL scale, while the control group included 53 patients who had no radiological changes in their knees (KL scale 0-I).

In the third phase of the study, fasting glucose levels and lipid panel were measured to assess metabolic parameters. The state reference laboratory at the Special Hospital for Rheumatic Diseases, Novi Sad, Serbia, was responsible for conducting all of the laboratory analyses. Metabolic syndrome (MetS) was identified based on the criteria established by the International Diabetes Federation (IDF) Consensus statements [23]. MetS was defined as the combination of central obesity (waist circumference of 80 cm or more) and at least two of the following four factors: elevated triglyceride levels (equal to or greater than 150 mg/dL or 1.7 mmol/L) or receiving specific treatment for this lipid abnormality; reduced levels of HDL cholesterol (less than 50 mg/dL or 1.29 mmol/L in females) or receiving specific treatment for this lipid abnormality; systolic blood pressure equal to or greater than 130 mm Hg or diastolic blood pressure equal to or greater than 85 mm Hg or undergoing treatment for previously diagnosed hypertension; and elevated fasting plasma glucose levels (equal to or greater than 100 mg/dL or 5.6 mmol/L) or previously diagnosed type 2 diabetes mellitus (T2DM).

**Figure 1:**
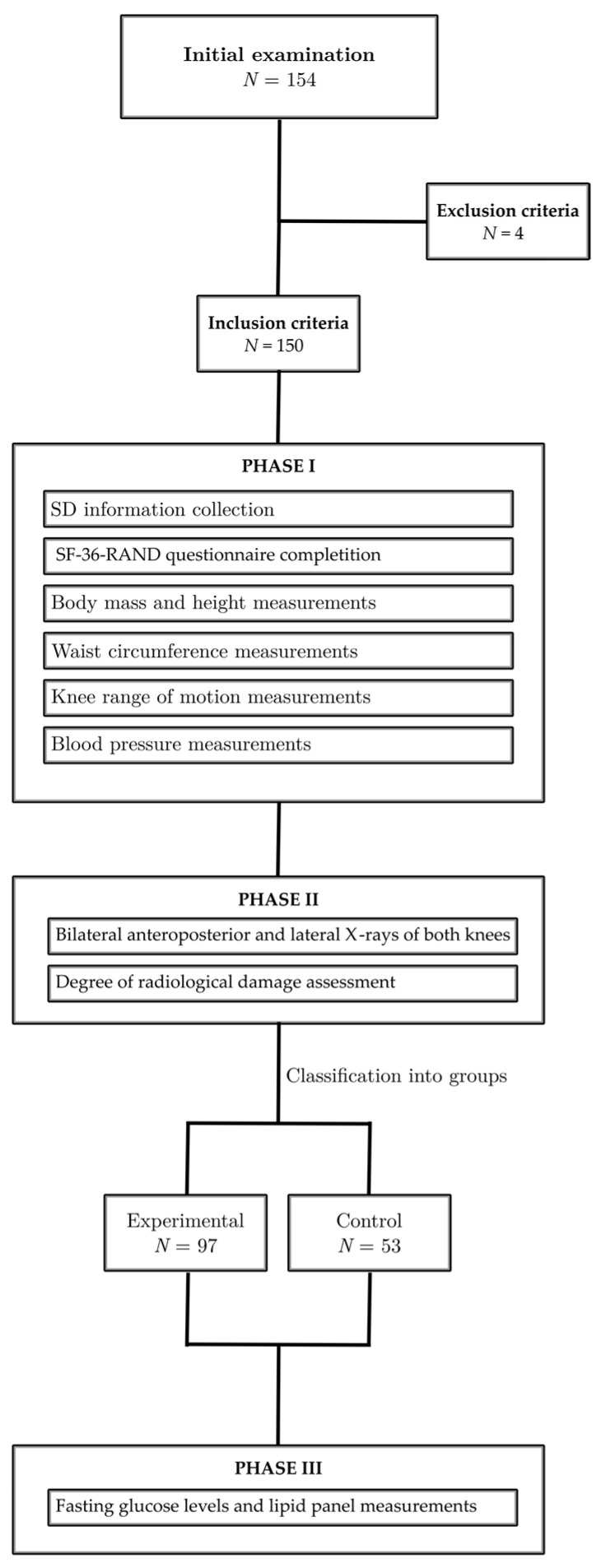
Cross-sectional design of the study.

In the extension, a flow diagram is presented, illustrating the cross-sectional design used for data collection, as well as the inclusion and exclusion criteria (Figure 1).

### 2.2. Assessment of Emotional Well-Being, Social Functioning, Physical Functioning, and Pain Level

The SF-36, RAND questionnaire comprises 8 subscales encompassing physical function, limitations in daily activities due to physical and emotional health, energy levels and fatigue, emotional well-being, social functioning, pain, and general health. Each subscale is scored on a scale of 0 to 100, with higher scores indicating greater levels of well-being and better overall health [20].

### 2.3. Evaluation of the Educational Attainment Level

Participants were divided into three groups according to their educational attainment level. The first group consisted of female patients with ≤8 years of schooling (primary education in Serbia lasts 8 years). The second group consisted of female patients with 8–12 years of schooling (secondary education in Serbia lasts 4 years). The third group consisted of respondents with more than 12 years of education (completed at least higher-education schooling).

### 2.4. Statistical Analysis

Considering the objectives of the study, the sample size was calculated with a confidence interval of 85% (consequently the power of the study was 85%), with a maximum error of 10% and a critical incidence value of 50%. As a result, a minimum of 52 respondents per group is required to meet these criteria. The statistical analysis involved examining differences among respondents using chi-squared tests and Mann–Whitney tests. All tests were conducted with a significance level of *p* < 0.05. To determine the relationship between the outcome variable (emotional well-being) and explanatory variables, a set of linear regression models (beta and 95% confidence interval—CI) was employed, considering both univariate and multivariate associations. The range of Cronbach’s alpha, which was between 0.796 and 0.885, indicates good internal consistency. All analyses were performed with IBM SPSS ver. 25 (IBM Corp., Armonk, NY, USA).

## 3. Results

The study included 150 women with an average age of 67 (IQR = 8.0). Women with KOA exhibited a higher body mass index (BMI) of 30.4 (IQR = 6.0) compared to the control group with a BMI of 28.6 (IQR = 6.6), with a statistically significant difference (*p* = 0.044). Furthermore, MetS was found to be more prevalent in the experimental group (81.4%) compared to the control group (58.5%), with a significant difference (*p* = 0.002). Among the participants, 23.3% had T2DM, and 75.3% had hypertension. The number of comorbidities among KOA participants ranged from 0 to 6, with an average of 3 (IQR = 1.0) (Table 1).

The study determined the reliability of the pain, social functioning, and emotional well-being subscales to be 0.796, 0.827, and 0.885, respectively. The average score obtained by the participants on the pain subscale was 47.8 (standard deviation = 19.6), on the social functioning subscale was 68.2 (standard deviation = 15.5), and on the emotional well-being subscale was 62.2 (standard deviation = 10.8). The descriptive statistics for the individual subscales of the SF-36 questionnaire are presented in Table 2.

Table 3 displays the univariate linear regression model involving the SF-36 RAND subscale of emotional well-being as the dependent variable. The impact of individual independent variables was examined, followed by their combined impact. The variables that individually explain emotional well-being are as follows: pain intensity (beta (β) = 0.21; 95% CI: 0.03–0.20; *p* < 0.01), social functioning (beta (β) = 0.47; 95% CI: 0.23–0.43; *p* < 0.001), physical functioning (beta (β) = 0.23; 95% CI: 0.04–0.21; *p* < 0.01), and education level (8–12 years: beta (β) = 0.25; 95% CI: 1.47–9.41; *p* < 0.01; >12 years: beta (β) = 0.27; 95% CI: 2.51–12.67; *p* < 0.01). Therefore, individuals with a better social functioning, improved physical functioning, and reduced pain tend to have better emotional well-being. Furthermore, respondents who have completed secondary school or higher education exhibit superior emotional well-being compared to those with primary education or less.

The multivariate model, which incorporates only the variables that demonstrated a statistically significant impact on the dependent variable in the univariate model, reveals that certain variables are associated with emotional well-being. Specifically, social functioning (beta (β) = 0.57; 95% CI: 0.27–0.53; *p* < 0.001) and education level (8–12 years: beta (β) = 0.21; 95% CI: 1.10–8.260; *p* < 0.05; >12 years: beta (β) = 0.21; 95% CI: 1.18–10.30; *p* < 0.05) demonstrate a connection with an enhanced emotional well-being (presented in Table 4). In summary, a better social functioning and a higher level of education are associated with improved emotional well-being.

## 4. Discussion

Our study indicates that pain intensity, social functioning, physical functioning, and education level are significant individual predictors of emotional well-being among KOA patients. However, the multivariate analysis reveals that among postmenopausal women with KOA, only social functioning and education level emerge as significant predictors of emotional well-being.

Advanced age, obesity, and physical inactivity are recognized as risk factors for various chronic diseases that are prevalent among individuals with KOA. Previous estimates indicate that a significant proportion of patients with OA, ranging from 59% to 87%, have been diagnosed with at least one additional chronic disease. Furthermore, as many as 31% of individuals with OA have the burden of five or more comorbidities [5,24]. Patients with OA typically experience an average of 2.6 comorbidities of moderate to severe intensity [5,24], which was consistent with the findings of our study (3 (IQR = 1.0)). Besides experiencing knee problems, individuals with KOA often encounter other chronic conditions, predominantly cardiovascular and pulmonary issues, as well as hypertension and diabetes [25]. Among the participants in our study, hypertension and T2DM were the prevailing ailments, and their occurrences are demonstrated in Table 1. The experimental and control groups were comparable in terms of the prevalence of hypertension and T2DM and there was no statistically significant distinction in the number of comorbidities between the observed groups (*p* = 0.696). Nonetheless, there was a significant difference in the number of subjects with MetS in the experimental group. The clinical significance of comorbidities in OA is acknowledged due to their potential impact on clinical practices, outcomes, treatment strategies, prognosis, health-related quality of life (HRQOL), and healthcare costs [25,26]. Calders et al. documented that an increased prevalence of additional health conditions exacerbates both pain and functional limitations among individuals suffering from hip and knee OA [11]. Contrary to our expectations, we were unable to establish a statistically significant correlation between the number of comorbidities and emotional well-being in patients with KOA. This discrepancy may be attributed to variations in the severity and duration of specific diseases, i.e., symptoms of the disease. In our previous pilot study [27], the number of comorbidities emerged as a significant predictor of emotional well-being, but the limited sample size could have introduced bias into the results.

As anticipated, the majority of participants in our study exhibited an elevated body mass. In a comprehensive meta-analysis encompassing 131 studies, it was determined that the average body mass index (BMI) among individuals with OA was 28.2 [9]. We observed an even higher BMI (30.4 (IQR = 6.0)) among subjects with radiographic KOA. This finding is not surprising, considering that our study specifically focused on participants with KOA and it is widely recognized that being overweight or obese significantly increases the risk of developing OA, particularly in the knee joint [28].

In the majority of studies, advancing age is linked to a decline in QOL [8]. It has been observed that younger individuals have greater expectations when it comes to their QOL, likely due to their higher efficiency in performing daily activities, work, and leisure [8,29]. We were unable to establish a correlation between emotional well-being and the age of the respondents. This can be attributed to the homogeneity of the group in terms of age and gender.

Utilizing a univariate linear regression model, we discovered that pain, social functioning, physical functioning, and education level serve as predictors for emotional well-being among patients with KOA. This aligns with the findings of Giesbrecht et al., who demonstrated a connection between psychological well-being and the ability to perform daily activities [30]. Additionally, Nazarinasab et al. reported that the extent of disability is associated with mental health [12]. Unlike knee ROM, physical functioning (evaluated using the SF-36-RAND questionnaire) emerged as a statistically significant predictor of emotional well-being. It has been suggested that a range of 100–110 degrees in knee flexion is essential for everyday activities [21], and we adopted these values as a reference. Nevertheless, there are several other activities, such as entering a bathtub, engaging in gardening, performing DIY tasks, or changing socks, that require a greater knee ROM [21,31].

In accordance with the outcomes of our study, Fonseca-Rodrigues et al. have demonstrated that the intensity of pain is linked to the extent of emotional distress experienced by patients with OA [9], while Laires et al., reported that levels of pain often correlate with reduced levels of satisfaction with treatment [32]. The existing literature suggests that mental health levels are inversely associated with knee pain, regardless of any observable changes in X-ray images [12]. Consistent with these findings, our study revealed that the severity of radiological knee damage does not predict emotional well-being among patients with KOA.

The educational attainment of patients with KOA significantly influences their QOL. It is widely acknowledged that there is a connection between education level and KOA, as individuals with lower education often engage in repetitive work tasks that heighten the risk of developing the condition. Moreover, having a lower educational level is associated with a decline in self-perceived QOL [8]. In a similar vein, our study’s findings indicate that respondents with a secondary school education or higher exhibit better emotional well-being when compared to those with only primary education or lower.

The correlation between social isolation and both mental and physical well-being has been previously emphasized [33]. Additionally, the importance of friends, family, and caregivers as vital providers of social support has been acknowledged, as they play a key role in helping individuals with OA maintain their independence and receive encouragement to participate in meaningful activities [34]. In our study, both univariate and multivariate regression models revealed that social isolation significantly predicts emotional well-being among individuals with KOA. It is well-established that pain and a decreased functionality can contribute to the risk of social isolation [33,35]. Moreover, individuals with OA face additional factors that increase the likelihood of social isolation, including anxiety, depression, kinesiophobia, physical inactivity, and a diminished self-efficacy, all of which impede their functional independence [33,36]. Additionally, the literature indicates that a considerable proportion of individuals aged 65 and older experience challenges related to low self-efficacy, depression, and a reduced sense of QOL and well-being, all of which compromise their personal independence [17,37].

Taking into account the higher susceptibility of women to developing knee and hand OA, particularly during the postmenopausal phase [9], we investigated the impact of postmenopausal duration on emotional well-being. However, this specific variable did not emerge as a statistically significant risk factor.

Positive emotions and overall well-being play a significant role in affecting the occurrence of various health conditions and diseases, as well as the risk of premature mortality [13]. Previously, there have been suggestions that well-being might influence physiological processes that are pertinent to the risk of developing arthritis or the manifestation of its symptoms [38]. Considering the association of OA with detrimental effects on healthcare utilization and escalating personal and societal expenses, [34] it becomes highly imperative to conduct an examination and understanding of the contributing factors that lead to a reduced emotional well-being. We believe that our research will provide valuable insights to enhance the management of KOA.

This study possesses some limitations. Firstly, the patients included in the study were not representative of the entire population of our country, but rather comprised patients from Vojvodina, a Serbian province, who were treated at a single healthcare facility. Secondly, important data on socioeconomic status, which is a significant factor and mediator for emotional well-being, were not collected during the enrollment process. However, this study was conducted prospectively, ensuring that every patient received a knee x-ray, and the extent of radiological damage was evaluated by a skilled radiologist using the KL scale and the Altman atlas. The data were collected by one trained researcher under consistent conditions throughout the study.

## 5. Conclusions

Our study revealed that poorer social functioning and lower educational attainment are associated with a higher risk of experiencing negative emotional well-being among patients with KOA. Typically, the treatment approach for KOA involves lifestyle modifications such as weight loss, exercise, physical therapy, pain-relieving medications, and, in some cases, surgical intervention. However, it is noteworthy that only a small proportion (estimated at 12%) of these interventions address the crucial aspect of social integration and support [38]. Therefore, it is imperative to emphasize the implementation of various strategies for social engagement, community participation, and specific forms of psychological support.

## Figures and Tables

**Table 1:** General characteristics of the respondents and frequent comorbidities.

	(ALL) *N* = 150	Experimental Group N = 97	Control Group N = 53	*p*-Value
Age (years), Me ± IQR (Min–Max)	67.0 ± 8.0 (60–75)	67.0 ± 7.0 (60–75)	65.0 ± 10.5 (60–75)	0.063 ^b^
BMI (kg/m^2^), Me ± IQR (Min–Max)	30.0 ± 6.4 (18.5- 47.3)	30.4 ± 6.0 (19.8–47.3)	28.6 ± 6.6 (18.5–42.1)	0.044 ^b^
Mets:				0.002 ^a^
Yes	110 (73.3%)	79 (81.4%)	31 (58.5%)	
No	40 (26.7%)	18 (18.6%)	22 (41.5%)	
T2DM:				0.882 ^a^
Yes	35 (23.3%)	23 (23.7%)	12 (22.6%)	
No	115 (76.7%)	74 (76.3%)	41 (77.4%)	
Hypertension:				0.246 ^a^
Yes	113 (75.3%)	76 (78.4%)	37 (69.8%)	
No	37 (24.7%)	21 (21.6%)	16 (30.2%)	
The number of comorbidities, Me ± IQR (Min–Max)	3.0 ± 1.0 (0–7)	3.0 ± 1.0 (0–6)	2.0 ± 1.0 (1–7)	0.696 ^b^

Note. ^a^ Chi-squared test. ^b^ Mann–Whitney.

**Table 2:** Descriptive statistics of SF-36 scale.

SF-36 Domains	Me	IQR	M	SD	Skewness	Kurtosis	K-S	α
Pain	45.0	32.5	47.8	19.6	0.057	−0.765	0.156 **	0.796
Social functioning	75.0	15.6	68.2	15.5	−0.477	0.066	0.265 **	0.827
Emotional well-being	64.0	12.0	62.2	10.8	−0.839	1.092	0.130 **	0.885

Note. ** *p* < 0.01. Me = Median. IQR = Inter-quartile range. M = Mean. SD = Std. Deviation. K-S = Kolmogorov–Smirnov test. α = Cronbach’s alpha.

**Table 3:** General characteristics and health status as determinants of emotional well-being, univariate linear regression.

	Univariate Linear Regression
Osteoarthritis (ref.: without osteoarthritis)	
Yes	−0.06 (−5.12–2.20)
Number of comorbidities (continuous)	−0.37 (−3.84–−1.37)
Range of motion (ref.: unsatisfactory functionality)	
Satisfactory functionality	−0.17 (−7.32–−0.37)
KL scale (continuous)	−0.10 (−2.42–0.55)
Duration of menopause (continuous)	−0.09 (−0.45–0.12)
SF36_pain (continuous)	0.21 (0.03–0.20) **
SF36_ social functioning (continuous)	0.47 (0.23–0.43) ***
SF36_ physical functioning (continuous)	0.23 (0.04–0.21) **
Age, years (continuous)	−0.02 (−0.44–0.32)
Lives alone (ref.: no)	
Yes	0.08 (−2.00–6.29)
Education level (ref.: ≤8)	
8–12	0.25 (1.47–9.41) **
>12	0.27 (2.51–12.67) **

Note. Values represent beta coefficients with corresponding 95% confidence intervals. ** *p* < 0.01; *** *p* < 0.001.

**Table 4:** The variables that individually explain emotional well-being, multivariate linear regression.

	Multivariate Linear Regression
SF36_pain (continuous)	−0.21 (−0.25–0.06)
SF36_social functioning (continuous)	0.57 (0.27–0.53) **
SF36_physical functioning (continuous)	0.13 (−0.13–0.14)
Education level (ref.: ≤8)	
8–12	0.21 (1.10–8.260) *
>12	0.21 (1.18–10.30) *

Note. Values represent beta coefficients with corresponding 95% confidence intervals. * *p* < 0.05; ** *p* < 0.001.

## Data Availability

Not applicable.
